# Food functionalities and bioactivities of protein isolates recovered from skipjack tuna roe by isoelectric solubilization and precipitation

**DOI:** 10.1002/fsn3.1470

**Published:** 2020-02-20

**Authors:** Jang Woo Cha, In Seong Yoon, Gyoon‐Woo Lee, Sang In Kang, Sun Young Park, Jin‐Soo Kim, Min Soo Heu

**Affiliations:** ^1^ Department of Seafood and Aquaculture Science/Institute of Marine Industry Gyeongsang National University Tongyeong Korea; ^2^ Department of Food and Nutrition/Institute of Marine Industry Gyeongsang National University Jinju Korea; ^3^ Research Center for Industrial Development of Seafood Gyeongsang National University Tongyeong Korea

**Keywords:** food functionality, physicochemical characterization, roe protein isolates, skipjack tuna

## Abstract

Four roe protein isolates (RPIs) from skipjack tuna were prepared using isoelectric solubilization (pH 11 and 12) and precipitation (pH 4.5 and 5.5) (ISP) at different pH points to evaluate their physicochemical and functional properties and in vitro bioactivities. Moisture (<6.3%) and protein (71%–77%) content were maintained. Sulfur, sodium, phosphorus, and potassium were the major elements, and glutamic acid and leucine were the prevalent amino acids (12.2–12.8 and 9.6–9.8 g/100 g protein, respectively) in RPIs. RPI‐1 showed the highest buffering capacity at pH 7–12. RPIs and casein showed similar water‐holding capacities. At pH 12, RPI‐1(pH 11/4.5) showed the highest solubility, followed by RPI‐3(pH 12/4.5), RPI‐2(pH 11/5.5), and RPI‐4(pH 12/5.5) (*p* < .05). Oil‐in‐water emulsifying activity indices of RPI‐1 and RPI‐3 significantly differed. At pH 2 and 7–12, pH‐shift treatment improved the food functionality of RPIs, which was superior to positive controls (casein and hemoglobin). RPI‐1 showed ABTS^+^ radical scavenging (102.7 μg/ml) and angiotensin‐converting enzyme inhibitory activities (44.0%).

## Graphical abstract

The physicochemical properties of skipjack tuna roe protein isolate (RPI) were determined, and the isolate was found to be useful as a protein source. These RPIs were superior to the positive controls in food functionalities and confirmed to be useful as raw materials in protein‐fortified foods. RPI also showed in vitro antioxidant and antihypertensive activities. Fish RPIs recovered using isoelectric solubilization, and precipitation can be used to supplement and enhance deficient nutrients in agricultural products. By partially replacing or adding RPI to traditional foods such as noodle, confectionery, and bakery‐ and surimi‐based products, agro‐seafood products with food functionality can be developed.

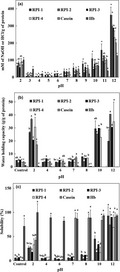

## 1 INTRODUCTION

Skipjack tuna (*Katsuwonus pelamis*) is consumed worldwide because of its abundance, nutritional value, and sensorial attributes (firm texture and flavorful flesh). This tuna is widely used as a raw material for sashimi and sushi in Korea and Japan (Lee, Park, et al., 2016). In 2015, 51,201 metric tons of skipjack tuna were canned, accounting for 57% of all canned products in Korea (KOSIS, 2016). The annual discard rate of fish processing byproducts has increased with increasing fish production. The fish processing industry generates large quantities of byproducts such as heads, skin, frames, roe, viscera, and scales (Narsing Rao, Prabhakara Rao, Satyanarayana, & Balaswamy, 2012), most of which are not used as sources of high value‐added products for human or animal consumption and are disposed of as waste. However, these byproducts are excellent sources of proteins (Lee, Lee, et al., 2016). Upcycling or reuse of byproducts has gained wide attention in the seafood processing industry owing to its economic effectiveness (Lee, Lee, et al., 2016). In recent years, numerous studies have examined the use of protein‐rich seafood byproducts as nutraceuticals or protein and lipid supplements (Kim, Yoon, Shim, & Lim, 2016).

Among fish byproducts, roes are nutritionally rich in essential fatty acids, minerals, and amino acids (Heu et al., 2006; Park et al., 2016). The production of caviar and roe products from other fish species has been examined previously (Bledsoe, G., Bledsoe, C., & Rasco, 2003).

Roe, a byproduct of fish processing (3.0%–30.0% depending on species), contains 11% albumin, 75% ovoglobulin, and 13% collagen (Sikorski, 1994) and is generally used as animal feed or in pet food preparation. The underutilized skipjack tuna roe (STR) requires processing methods for conversion to more marketable and acceptable forms, such as protein concentrates, isolates, and extracts. Previous studies characterized the ingredients, chemical composition (Heu et al., 2006; Lee, Lee, et al., 2016), fractions, and characteristics of the protease inhibitors (Kim et al., 2013), concentrates (Park et al., 2016; Yoon, Lee, et al., 2018), and protein isolates (Lee, Park, et al., 2016) in fish roes.

Isoelectric solubilization and precipitation (ISP) promotes the selective pH‐induced water solubility of muscle proteins, segregates lipids, and removes materials unfit for human consumption, such as bones, scales, and skin (Hultin & Kelleher, 1999). ISP is used for protein recovery from inaccessible sources such as fish (Chen & Jaczynski, 2007), chicken (Tahergorabi, Beamer, Matak, & Jaczynski, 2011), and beef (Mireles, Gomez, & James, 2002) processing byproducts; additionally, the fat content in the recovered protein isolate is significantly reduced. A major technology was developed for feasible extraction of functional protein isolates from low‐value raw materials (dark muscle fish and fatty fish) and seafood processing byproducts (fish cutoffs and fish frame) (Nolsoe & Undeland, 2009). Proteins recovered from the ISP process exhibit desirable functional properties and may have better gelling properties than proteins obtained by conventional surimi processing (Kristinsson, Theodore, Demir, & Ingadottir, 2005). Proteins are the basic functional components of processed foods and determine the textural and nutritional properties and contribute to the quality and sensorial attributes of foods (Mohamed, Xia, Issoufou, & Qixing, 2009; Park et al., 2016). No studies have examined the protein isolate preparation of skipjack tuna roe or its food functional characteristics and bioactivity. The purpose of this study was to investigate the physicochemical and functional characteristics of protein isolates recovered from skipjack tuna roe using the ISP process, as well as their antioxidative and antihypertensive bioactivities.

## 2 MATERIALS AND METHODS

### 2.1 Chemicals

2,2′‐Azino‐bis (3‐ethylbenzothiazoline‐6‐sulfonic acid) ammonium salt (ABTS), casein, hemoglobin, hippuryl‐his‐leu acetate salt (HHL), lung acetone powder from rabbit, mushroom tyrosinase, potassium persulfate, and sodium L‐tartrate were purchased from Sigma‐Aldrich. 3,4‐Dihydroxy‐L‐phenylalanine (L‐DOPA) was purchased from Acros Organics. Hydrochloric acid (1 M) and sodium hydroxide (1 M) were purchased from Yakuri Pure Chemicals Co., Ltd.. Folin–Ciocalteu reagent and acetic acid were purchased from Junsei Chemical Co., Ltd. Sodium dodecyl sulfate (SDS) and glycine were purchased from Bio Basic, Inc. Soybean oil was purchased from Ottogi Co., Ltd. All other reagents were of analytical grade.

### 2.2 Sample

Skipjack tuna roe was obtained from Dongwon F & B Co., Ltd. (Changwon, Korea), stored at −70°C in sealed polyethylene bags, and transferred to the laboratory. Frozen roe was partially thawed for 24 hr at 4°C and then cut into small pieces to 2.0 cm thickness and minced with a food grinder. Minced roe was frozen at −20°C until use.

### 2.3 Preparation of roe protein isolates (RPIs)

Roe protein isolates were prepared as described by Lee, Lee, et al. (2016). The frozen minced roe was homogenized with deionized distilled water (DDW) at a 1:6 (w/v) ratio using a homogenizer. The homogenate was adjusted to pH 11 and 12 with 2N NaOH. Solubilization was allowed to occur at 4°C for 1 hr, followed by centrifugation at 12,000 × *g* and 4°C for 30 min. After centrifugation, two alkali‐soluble (pH 11 and 12) supernatant fractions were collected.

To prepare the RPIs from the alkali‐soluble fractions by acid precipitation, the pH of the fractions was readjusted to pH 4.5 and 5.5 (similar to the isoelectric point of fish proteins) by adding 2N HCl. The suspensions were centrifuged at 12,000 × *g* and 4°C for 30 min. Precipitates obtained after ISP were washed with DDW and centrifuged at 12,000 × *g* and 4°C for 30 min to remove the NaCl. After centrifugation, the washed RPIs were lyophilized and labeled as RPI‐1 (pH 11/4.5), RPI‐2 (pH 11/5.5), RPI‐3 (pH 12/4.5), and RPI‐4 (pH 12/5.5). These RPIs were stored at −20°C until further use.

### 2.4 Physicochemical properties

#### 2.4.1 Proximate composition

The proximate composition was determined by the AOAC method (AOAC, 2005). Moisture content (950.46) was determined by placing a 0.5‐g sample in an aluminum pan and drying in a forced air convection oven at 105°C until a constant mass was reached. The ash content (920.153) was determined by charring approximately 0.2 g sample in a ceramic crucible over a hot plate and then heating in a muffle furnace at 550°C until a constant mass was achieved. The total crude protein content (928.08) was determined by the semimicro Kjeldahl method. Total lipid content (960.39) was determined by the Soxhlet extraction method. Sample (5 g) was extracted with dimethyl ether for 30 min at a drip rate of 10 ml/min. The total lipid content was determined by gravimetry and expressed as a percentage.

#### 2.4.2 Protein concentration

The protein concentration of the samples was determined as described by Lowry, Rosebrough, Farr, and Randall (1951) using bovine serum albumin as a standard.

#### 2.4.3 Total amino acid content

The samples were hydrolyzed by 6 N HCl at 110°C for 24 hr on a heating block and filtered using a vacuum filtration device. Amino acids were quantified with an amino acid analyzer (Biochrom 30, Biochrom Ltd.) using sodium citrate buffers (pH 2.2) in step gradients. The results are reported in milligrams of amino acid per 100 g protein.

#### 2.4.4 Mineral analysis

The mineral content of the samples was determined by inductively coupled plasma optical emission spectrophotometry (Optima 4300 DV, PerkinElmer). The samples were dissolved in 10 ml of 70% (v/v) nitric acid and heated on a hot plate until digestion was complete. The volume of the samples (in duplicates) was made up to 100 ml with 2% nitric acid in a volumetric flask. The mineral concentration was calculated and expressed as milligrams per 100 g sample.

#### 2.4.5 Color value

The samples were equilibrated to 20 ± 2°C for 2 hr prior to Hunter color measurement. Color values were determined using a color meter (ZE‐2000 Nippon Denshoku Inc.). The colorimeter was calibrated using a standard plate (L* [lightness] = 96.82, a* [redness] = −0.35, b* [yellowness] = 0.59) supplied by the manufacturer. The values of the Commission Internationale d’Eclairage of France (CIE) color system using tri‐stimulus color values (L*, a*, and b*) were determined. Whiteness was calculated using the following equation:

$$Whiteness=\text{100}-\sqrt{{\left(100-L^{*}\right)}^{2}+a^{*2}+b^{*2}}$$

### 2.5 SDS–polyacrylamide gel electrophoresis (SDS‐PAGE)

The molecular weight distribution of proteins was investigated by SDS‐PAGE, which was performed according to the method of Laemmli (1970). Briefly, 20 mg sample was solubilized in 5 ml of 5% SDS. The protein solution was then mixed at a 4:1 (v/v) ratio with SDS‐PAGE sample treatment buffer (pH 6.8) and boiled at 100°C for 3 min. Samples (20 µg protein) were loaded into a 10% Mini‐PROTEAN^®^ TGX™ precast gel and electrophoresed at a constant current of 10 mA using a Mini‐PROTEAN^®^ tetra cell (Bio‐Rad).

### 2.6 Protein functionalities

#### 2.6.1 Buffer capacity

Buffer capacity was measured as described by Park et al. (2016). The sample (300 mg) was dispersed in 30 ml distilled water, and measured volumes of 0.5 M NaOH and HCl were added in small increments. The corresponding changes in pH in alkaline and acidic ranges were recorded. The amounts of base and acid added were plotted against the pH, and the buffer capacity in each range was expressed as the mean value of NaOH or HCl (in millimolar) required per gram sample to induce a unit change in pH.

#### 2.6.2 Water‐holding capacity (WHC)

The WHC of samples was measured as described by Park et al. (2016). The sample (300 mg) was mixed with 30 ml DDW in a 50‐ml centrifuge tube. The mixture was thoroughly vortexed for 10 min at 20 ± 2°C and centrifuged at 12,000 × *g* for 20 min at 4°C. WHC was determined from the difference in masses and expressed as grams of water absorbed per gram protein.

$$\text{WHC (g}/\text{g protein)}=\frac{\text{Mass}\;\;\text{of}\;\text{pellet}\;(g)-\text{Mass}\;\text{of}\;\text{sample}\;\text{(g)}}{\text{Mass}\;\text{of}\;\text{sample}\;(g)\times C}$$

where C is protein concentration (%).

#### 2.6.3 Protein solubility

The protein solubility of samples was determined according the method of Park et al. (2016). First, 300 mg sample was dispersed in 30 ml DDW and the pH was adjusted to 2–12 with 2N HCl and 2N NaOH. The mixture was stabilized at room temperature for 30 min prior to centrifugation at 12,000 *g* for 20 min. Protein content in the supernatant and total protein content in the sample were determined by Lowry's method after solubilizing the sample in 2N NaOH. Protein solubility was calculated as follows:

$$\text{Solubility}\;(\%)=\frac{\text{Protein content in supernatant}}{\text{Total protein content in sample}}\times 100$$

Each measurement was replicated at least five times, and the results were expressed as the means ± *SD*.

#### 2.6.4 Foaming capacity (FC) and foam stability (FS)

Foaming capacity (FC) and foam stability (FS) of the sample solution (1%, w/v) were determined as described by Park et al. (2016) with slight modifications. Ten milliliters of 1% (w/v) sample solution was transferred to a 25‐ml volumetric cylinder. The solution was homogenized (Polytron^®^ PT 1200E, Kinematica AG) at 12,500 rpm for 1 min at 20 ± 2°C. The sample was allowed to stand for 0, 15, 30, and 60 min, and FC and FS were calculated using the following equations:

$$\text{Foaming capacity (}\%)=\frac{\text{VT}}{\text{V0}}\times 100$$

$$\text{Foam stability (}\%)=\frac{\left(\text{Ft}/\text{Vt}\right)}{\left(\text{FT}/\text{VT}\right)}\times 100$$

where VT is the total volume after whipping, V_0_ is the initial volume, and Ft and Vt are the total foam and total volume, respectively, after standing at room temperature for different durations (t = 15, 30, and 60 min).

#### 2.6.5 Emulsifying properties

The emulsifying activity index (EAI) and emulsion stability index (ESI) were determined as described by Park et al. (2016). Soybean oil (Ottogi Co., Ltd.) and 1% (w/v) sample at a 1:3 (v/v) ratio were homogenized at a rate of 12,500 rpm for 1 min. An aliquot of the emulsion (50 μl) was pipetted from the bottom of the volumetric cylinder 0 and 10 min after homogenization and mixed with 5 ml of 0.1% SDS solution. The absorbance of the mixture was measured at 500 nm (UV‐2900, Hitachi).

The absorbances measured immediately (A_0min_) and at 10 min (A_10min_) after emulsification were used to calculate EAI and ESI as follows:

$${\text{EAI (m}}^{2}/\text{g)}=\frac{2\times 2.303\times A\times DF}{l\times \varphi \times C}\times 100$$

where A is the absorbance at 500 nm, DF is the dilution factor (100), *l* is the path length of the cuvette (1 cm), φ is the oil volume fraction (0.25), and C is the protein concentration in the aqueous phase (g/ml).

$$\text{ESI (}\min)=\frac{A0\times \Delta t}{\Delta A}$$

where ΔA = A_0min_–A_10min_ and Δt = 10 min. A_0min_ and A_10min_ are the absorbance values measured at 0 and 10 min after emulsification, respectively.

### 2.7 Antioxidative and antihypertensive activity

#### 2.7.1 ABTS^+^ radical scavenging activity

ABTS^+^ radical scavenging activity was determined using the ABTS assay with slight modification based on the method of Binsan et al. (2008). Fresh ABTS^+^ solutions in ethanol were prepared for each assay. Samples (1 ml) were mixed with 3 ml ABTS^+^ solution and incubated at room temperature for 30 min in the dark. Absorbance was measured at 734 nm with a spectrophotometer. The IC_50_ value was defined as the concentration required to scavenge 50% of ABTS^+^ radical.

Absorbance was measured immediately (A_734_), and ABTS^+^ radical scavenging activity was calculated as follows:

$${\text{ABTS}}^{+}\;\text{radical scavenging activity (}\%)=\frac{\left({\text{Control}}_{734}-{\text{Sample}}_{734}\right)}{{\text{Control}}_{734}}\times 100$$

where control_734_ is the absorbance of the same reaction system without the sample.

#### 2.7.2 Tyrosinase inhibitory activity

The tyrosinase inhibitory activity was determined as described by Iida et al. (1995) with some modifications. Briefly, 900 μl (50 U/ml reaction mixture) mushroom tyrosinase was preincubated with the sample in 50 mM phosphate buffer (pH 6.8) for 30 min at room temperature. Next, 300 μl 10 mM L‐DOPA was added to the reaction mixture, and enzyme activity was monitored at room temperature by measuring the change in absorbance at 475 nm (UV‐2900, Hitachi) for 30 min at 1‐min intervals, which corresponded to the formation of dopachrome.

Samples without inhibitor were used as controls. The percentage inhibition of enzyme activity by the active compounds was calculated as follows:

$$\text{Tyrosinase inhibitory activity (}\%)=\frac{({\text{Control}}_{475}-{\text{Sample}}_{475)}}{{\text{Control}}_{475}}\times 100$$

where control_475_ is the absorbance of a reaction system without the sample.

#### 2.7.3 Angiotensin‐converting enzyme (ACE) inhibitory activity

Angiotensin‐converting enzyme inhibitory activity was estimated as described by Cushman and Cheung (1971) with slight modifications. ACE was extracted from 5 g lung acetone powder from rabbit with 100 ml 0.05 M sodium borate buffer (pH 8.3) containing 300 mM NaCl. A mixture of 100 μl sample, 50 μl ACE extracts and 50 μl 0.05 M sodium borate buffer (pH 8.3), was preincubated at room temperature for 30 min before incubation with 50 μl substrate (5 mM HHL in 0.05 M sodium borate buffer, pH 8.3) for 60 min at 37°C in a water bath. The reaction was terminated by adding 250 μl 1N HCl. The resulting hippuric acid was extracted with 1.5 ml ethyl acetate. After centrifugation (1,890 × *g*, 10 min, 4°C), 1.0 ml of the upper layer was transferred into a test tube and evaporated at 100°C for 1 hr in a heating block. The hippuric acid was dissolved in 1.0 ml distilled water, and absorbance was measured at 228 nm with a UV spectrophotometer. Absorbance was measured immediately (A_228_) and used to calculate the ACE inhibitory activity as follows:

$$\text{ACE inhibitory activity (}\%)=\left[1-\frac{{\text{Sample}}_{228}-{\text{Control Blank}}_{228}}{{\text{Control}}_{228}-{\text{Control Blank}}_{228}}\right]\times 100$$

where the sample blank is the absorbance of the inactivated sample before adding HHL and control blank is the absorbance of the inactivated control before adding HHL.

### 2.8 Statistical analysis

All analyses were performed in triplicate. Data were averaged, and the *SD* was calculated. Data were analyzed by analysis of variance using SPSS 12.0 K software (SPSS, Inc.) for Windows. Mean comparisons were performed using the multiple range Duncan's test (*p* < .05).

## 3 RESULTS AND DISCUSSION

### 3.1 Physicochemical properties

#### 3.1.1 Proximate composition

Roe protein isolates were recovered from STR by ISP. The proximate composition, mineral content, and Hunter's color values of the RPIs and positive controls (casein and hemoglobin) are shown in Table 1. Freeze‐dried STR (FDSTR) contained 6.2 ± 0.9% moisture, 71.1 ± 0.7% protein, 15.2 ± 1.3% lipid, and 7.2 ± 0.1% ash. FDSTR yield and protein yield were 26.7 and 19.0 g/100 g of STR, respectively. The yields of RPIs produced from STR by ISP differed slightly by 10.3%–13.0%. The recovery of RPIs from STR by ISP was 54.2%–68.4%. This low protein recovery may be attributed to the presence of alkaline insoluble proteins that did not dissolve during alkaline solubilization (Lee, Lee, et al., 2016; Yoon, Kang, et al., 2018). The RPI‐2 showed the highest protein content (76.8 ± 0.3%), followed by RPI‐4 (75.4 ± 0.4%), RPI‐1 (71.4 ± 0.8%), and RPI‐3 (70.8 ± 0.7%). However, these values were lower than those of the positive controls (85.5 ± 0.0% for casein, 94.4 ± 0.4% for hemoglobin). The protein content of RPIs was higher than those reported for fish protein powders from arrowtooth flounder and herring (Sathivel et al., 2004). The protein content in fish eggs (Sathivel, Yin, Bechtel, & King, 2009) and surimi (Huda, Abdullah, & Babji, 2001) was similar to those in the RPIs. Lipid (13.8 ± 2.5–15.6 ± 1.0%) and ash content (1.8 ± 0.6–3.6 ± 0.1%) in the RPIs were lower than those in FDSTR (*p* < .05). This reduction in lipid and mineral content occurred because of mineral and fat migration into the processed water during ISP (Lee et al., 2017; Lee, Lee, et al., 2016). The protein content of RPI‐2 and RPI‐4 precipitated at pH 5.5 after alkaline solubilization was significantly higher than that of RPI‐1 and RPI‐3 precipitated at pH 4.5 (*p* < .05). In contrast, the total yield (10.3–10.9 g/100 g) and protein yield (7.8–7.9 g/100 g) of RPI‐1 and RPI‐2 were significantly lower than those of RPI‐3 and RPI‐4 (*p* < .05). Thus, alkali solubilization at pH 12 was better than that at pH 11, while acid precipitation did not affect protein yield (*p* < .05).

**Table 1:** Proximate composition and mineral content of roe protein isolates (RPIs) recovered from skipjack tuna roe (STR) by isoelectric solubilization and precipitation process

		FDSTR	RPI−1	RPI−2	RPI−3	RPI−4	Casein	Hb
Yield†, † (g)		26.7	10.9	10.3	13.0	12.2		
Protein yield‡, ‡ (g)		19.0	7.8	7.9	9.2	9.2		
Moisture (%)		6.2 ± 0.9^a^	5.7 ± 0.3^a^	4.5 ± 0.1^bc^	6.3 ± 0.1^a^	4.9 ± 0.3^b^	4.0 ± 0.2^c^	2.0 ± 0.0^d^
Protein (%)		71.1 ± 0.7^e^	71.4 ± 0.8^e^	76.8 ± 0.3^c^	70.8 ± 0.7^e^	75.4 ± 0.4^d^	85.5 ± 0.0^b^	94.4 ± 0.4^a^
Lipid (%)		15.2 ± 1.3^a^	15.6 ± 1.0^a^	14.0 ± 2.7^a^	14.7 ± 2.0^a^	13.8 ± 2.5^a^	ND	ND
Ash (%)		7.2 ± 0.1^a^	3.6 ± 0.1^b^	1.8 ± 0.6^c^	3.3 ± 0.4^b^	2.0 ± 0.1^c^	ND	ND
Minerals (mg/100 g)	K	1,097.4 ± 9.0^a^	18.4 ± 3.1^e^	70.3 ± 0.6^c^	25.8 ± 2.9^e^	45.2 ± 2.1^d^	912.0 ± 12.3^b^	71.4 ± 3.0^c^
	Na	615.2 ± 1.4^ab^	70.1 ± 0.6^b^	113.9 ± 3.2^b^	147.0 ± 2.1^b^	166.9 ± 1.1^b^	706.3 ± 10.9^a^	212.6 ± 1.8^b^
	Mg	66.9 ± 0.1^a^	1.7 ± 0.1^e^	7.1 ± 0.1^c^	2.3 ± 0.1^d^	7.6 ± 0.2^b^	ND	ND
	Zn	39.3 ± 0.3^c^	8.3 ± 0.0^d^	46.5 ± 0.5^b^	7.6 ± 0.1^d^	51.4 ± 0.9^a^	ND	ND
	Ca	36.3 ± 0.2^b^	4.8 ± 0.1^d^	15.6 ± 0.3^cd^	7.8 ± 0.2^d^	15.5 ± 0.2^cd^	987.2 ± 17.3^a^	21.2 ± 0.2^c^
	Fe	9.2 ± 0.2^de^	10.8 ± 0.2^d^	16.4 ± 0.3^b^	12.1 ± 0.2^c^	16.4 ± 0.2^b^	4.8 ± 0.0^f^	250.1 ± 1.8^a^
	P	247.9 ± 1.5^a^	111.9 ± 0.6^e^	115.5 ± 1.0^d^	120.3 ± 1.0^c^	131.5 ± 1.4^b^	34.7 ± 0.4^f^	29.1 ± 0.3^g^
	S	974.3 ± 57.7^b^	482.5 ± 89.5^ef^	683.7 ± 83.6^cd^	573.7 ± 72.1^de^	782.1 ± 47.2^c^	1984.1 ± 3.2^a^	442.0 ± 46.2^f^
Color values	L*	59.2 ± 0.3^a^	55.0 ± 0.2^b^	52.2 ± 0.5^c^	52.6 ± 0.5^c^	51.9 ± 0.4^c^		
	a*	6.8 ± 0.1^a^	4.1 ± 0.0^e^	5.3 ± 0.1^b^	4.3 ± 0.1^d^	5.1 ± 0.1^c^		
	b*	21.2 ± 0.0^a^	16.2 ± 0.2^d^	17.2 ± 0.2^b^	16.8 ± 0.2^c^	17.3 ± 0.1^b^		
	ΔE	43.5 ± 0.3^d^	44.7 ± 0.3^c^	48.0 ± 0.4^a^	47.3 ± 0.4^b^	48.3 ± 0.3^a^		
	Whiteness	53.6 ± 0.3^a^	52.0 ± 0.1^b^	48.9 ± 0.4^d^	49.6 ± 0.4^c^	48.6 ± 0.3^d^		

Note

Values are mean ± *SD* of triplicate determinations. Means with different letters within the same row are significantly different at *p* < .05 by Duncan's multiple range test.

FDSTR, freeze‐dried skipjack tuna roe; Hb, hemoglobin. ND: not determined; RPI‐1 and RPI‐2, roe protein isolate adjusted to pH 4.5 and 5.5, respectively, after alkaline solubilization at pH 11; RPI‐3 and RPI‐4, roe protein isolate adjusted to pH 4.5 and 5.5, respectively, after alkaline solubilization at pH 12.

^†^ Yield is weight (g) of each sample obtained from 100 g of raw STR.

^‡^ Protein yield (g) = yield × protein (%).

#### 3.1.2 Minerals

The mineral content of FDSTR, RPIs, and positive controls is shown in Table 1. The major minerals in FDSTR were potassium (1,097.4 ± 9.0 mg/100 g), sulfur (974.3 ± 57.7 mg/100 g), sodium (615.2 ± 1.4 mg/100 g), and phosphorus (247.9 ± 1.5 mg/100 g). The predominant minerals in casein were sulfur (1,984.1 ± 3.2 mg/100 g), calcium (987.2 ± 17.3 mg/100 g), and sodium (706.3 ± 10.9 mg/100 g), and their levels were higher than those in FDSTR. Sulfur (442.0 ± 46.2 mg/100 g), iron (250.1 ± 1.8 mg/100 g), and sodium (212.6 ± 1.8 mg/100 g) were the major minerals in hemoglobin, and their levels were lower than those in FDSTR, except for iron. The sulfur and potassium content in RPIs were 482.5 ± 89.5–782.1 ± 47.2 and 18.4 ± 3.1–70.3 ± 0.6 mg/100 g, respectively, which were significantly lower than those in FDSTR. The sodium (70.1 ± 0.6–166.9 ± 1.1 mg/100 g) and phosphorus (482.5–782.1 mg/100 g) levels in the RPIs were also significantly lower than those in FDSTR (*p* < .05), while magnesium and calcium levels showed similar trends. However, the sodium content in the RPIs was lower than those in crab (266.8 mg/100 g) (Gokoglu & Yerlikaya, 2003), hoki (620.0 mg/100 g) (Gokoglu, Yerlikaya, & Cengiz, 2004), and fish‐based dishes (222.8 mg/100 g) (Martinez‐Valverde, Periago, Santaella, & Ros, 2000). Phosphorus content in the RPIs was higher than that in rainbow trout (337.8 mg/100 g) (Gokoglu et al., 2004) and European perch (215–230.0 mg/100 g) (Orban et al., 2007). Overall, the total mineral content in RPI‐1 (pH 11/4.5) and RPI‐3 (pH 12/4.5) was significantly lower than those in RPI‐2 (pH 11/5.5) and RPI‐4 (pH 12/5.5) (*p* < .05). Acid precipitation at pH 4.5 during ISP was found to be effective for removing minerals (potassium, sodium, sulfur, and phosphorus) except for iron. Therefore, most minerals in FDSTR migrated into the processed water during ISP (Lee et al., 2017; Lee, Lee, et al., 2016).

#### 3.1.3 Color values

The color properties of FDSTR and the RPIs are shown in Table 1. The L* value of FDSTR (59.2) was higher than those of the RPIs (51.9 ± 0.4–55.0 ± 0.2), and the overall brightness of the RPIs obtained after ISP was decreased (*p* < .05). RPI‐1 and RPI‐3 showed lower a* values (4.1 ± 0.0 and 4.3 ± 0.1, respectively) than the other RPIs. Additionally, the b* value and color difference (ΔE) showed similar trends, confirming that acid precipitation at pH 4.5 during ISP caused color fading. The whiteness of RPI‐1 (52.0 ± 0.1) and RPI‐3 (49.6 ± 0.4) was significantly higher than those of the other RPIs (*p* < .05), but lower than that of FDSTR (53.6 ± 0.3) (*p* < .05). The RPIs obtained after ISP were also fainter than the dark brown FDSTR. The differences in color values may have resulted from the fractionation effect of the pigment induced by ISP. The relatively low L* and high a* values indicate that the product was brown. The color and whiteness of the fish protein isolates may be partially affected by the presence of connective tissues, which increase brightness, whereas lipid retention can affect yellowness. Additionally, the yellow‐brownish color of the product may be attributed to the deposition of heme proteins, which may affect redness, or to the denaturation and oxidation of hemoglobin (Kristinsson & Rasco, 2000).

#### 3.1.4 Total amino acids

The total amino acid composition (g/100 g protein, %) of FDSTR, RPIs, and positive controls is shown in Table 2. The protein content of all samples ranged from 75.8% to 96.3% based on the dry weight. The RPIs contained high levels of glutamic acid, aspartic acid, leucine, and lysine, which accounted for 12.2%–12.8%, 8.8%–9.0%, 9.6%–9.8%, and 8.4%–8.8% of the total amino acids, respectively. Intarasirisawat, Benjakul, and Visessanguan (2011) reported that the major nonessential amino acids (NEAA) in defatted tuna roes were glutamic acid and aspartic acid, while leucine and lysine were the predominant essential amino acids (EAA), which agrees with our results. The lysine content in the RPIs was higher than that in *Channa striatus* (6.94%) and *Lates calcarifer* (6.86%) (Narsing Rao et al., 2012). The EAAs in the RPIs ranged from 52.9% to 53.8%, which was higher than that in FDSTR (50.1%). The EAA/NEAA ratio in the RPIs ranged from 1.12 to 1.17, which was higher than that in casein (0.92) and slightly lower than that in hemoglobin (1.33). The protein nutritional value of the RPIs was improved by 12%–17% compared with that of FDSTR because of the decrease in NEAA content during ISP. The amino acid compositions of FDSTR and the RPIs were similar to those of mullet, cod, pollock, chinook salmon roe, and yellowfin tuna roe isolates (Bledsoe et al., 2003; Lee, Park, et al., 2016). The hydrophobic amino acid content in the RPIs and positive controls was 45.0%–45.9% and 44.6%–49.5%, respectively. The significantly lower content of proline and glycine in the RPIs compared with in FDSTR may be because of the precipitation of collagenous material (alkaline insolubles) during alkali solubilization (Lee, Lee, et al., 2016; Yoon, Kim, & Heu, 2018). However, the hydrophobic amino acid content, apart from glycine and proline, in the RPIs was significantly higher than that in FDSTR (*p* < .05). Overall, the EAA content in FDSTR and the RPIs were higher than those in casein (47.9%) but lower than those in hemoglobin (57.1%). Therefore, the concentrates and isolates from fish roe differed in composition based on the habitat environment, although fish roe proteins showed excellent nutritional attributes (Lee, Lee, et al., 2016; Park et al., 2016). Water‐holding or fat‐binding capacities are functional features that are closely related to texture by interactions between water, oil, or other food components. These functional properties are affected by the degree of exposure of hydrophilic (Asp, Glu, Lys, and Arg) and hydrophobic (Tyr, Ile, Val and Phe etc.) amino acid residues within the protein (Jellouli et al., 2011).

**Table 2:** Amino acid composition (g/100 g protein) of protein isolates recovered from skipjack tuna roe (RPIs) using isoelectric solubilization and precipitation process

Amino acid	FDSTR	RPIs				Casein	Hb
		RPI−1	RPI−2	RPI−3	RPI−4		
Protein content (%)†, †	75.8	75.7	80.4	75.6	79.3	89.1	96.3
Thr	5.1^a^	4.8^bc^	4.9^b^	4.8^bc^	4.9^b^	3.9^d^	4.7^c^
Val‡, ‡	6.2^d^	6.3^cd^	6.1^e^	6.4^c^	6.4^c^	7.3^b^	10.2^a^
Met‡, ‡	2.8^b^	3.1^a^	3.1^a^	3.1^a^	3.2^a^	1.3^c^	0.0^d^
ILe‡, ‡	5.1^d^	6.3^ab^	6.2^b^	6.4^a^	6.4^a^	5.7^c^	0.8^e^
Leu‡, ‡	8.3^e^	9.8^b^	9.7^bc^	9.6^c^	9.8^b^	9.1^d^	13.3^a^
Phe‡, ‡	4.1^d^	4.8^bc^	4.7^c^	4.8^bc^	4.9^b^	5.0^b^	7.6^a^
His	3.5^b^	2.9^c^	3.0^c^	2.9^c^	3.0^c^	2.9^c^	6.4^a^
Lys	8.4^d^	8.7^b^	8.6^c^	8.4^d^	8.8^b^	8.3^d^	10.3^a^
Arg	6.6^a^	6.6^a^	6.6^a^	6.4^b^	6.5^ab^	4.4^c^	3.8^d^
EAAs (%)	50.1	53.3	52.9	52.9	53.8	47.9	57.1
Asp	9.0^b^	8.8^c^	8.8^c^	9.0^b^	9.0^b^	8.2^d^	11.2^a^
Ser	6.0^a^	5.9^a^	5.7^b^	5.6^b^	5.4^c^	4.0^e^	4.4^d^
Glu	13.2^b^	12.2^e^	12.4^d^	12.8^c^	12.5^d^	22.1^a^	9.3^f^
Pro‡, ‡	5.8^b^	4.6^c^	4.2^d^	4.6^c^	4.1^de^	10.0^a^	4.0^e^
Gly‡, ‡	4.7^a^	3.6^b^	3.6^b^	3.7^b^	3.7^b^	2.4^c^	4.6^a^
Ala‡, ‡	6.8^c^	7.4^b^	7.4^b^	7.2^bc^	7.2^bc^	3.8^d^	9.0^a^
Cys	1.1^a^	0.9^b^	1.2^a^	0.9^b^	0.8^b^	0.5^c^	0.1^d^
Tyr	3.4^b^	3.3^b^	3.8^a^	3.4^b^	3.4^b^	1.2^c^	0.3^d^
NEAAs (%)	49.9	46.7	47.1	47.1	46.2	52.1	42.9
Total (%)	100.0	100.0	100.0	100.0	100.0	100.0	100.0
EAAs/NEAAs	1.00	1.14	1.12	1.12	1.17	0.92	1.33
HAAs (%)	43.8	45.9	45.0	45.8	45.7	44.6	49.5

Note

Values with different letters within the same row are significantly different at *p* < .05 by Duncan's multiple range test. Data are means of duplicate determination.

Abbreviations: EAAs, essential amino acids; HAAs, hydrophobic amino acids. FDSTR, freeze‐dried skipjack tuna roe; Hb, haemoglobin; NEAAs, nonessential amino acids; RPI‐1 and RPI‐2, roe protein isolates adjusted to pH 4.5 and 5.5, respectively, after alkaline solubilization at pH 11; RPI‐3 and RPI‐4, roe protein isolates adjusted to pH 4.5 and 5.5, respectively, after alkaline solubilization at pH 12.

^†^ Based on dry basis.

^‡^ Hydrophobic amino acid.

### 3.2 SDS–PAGE

**Figure 1:**
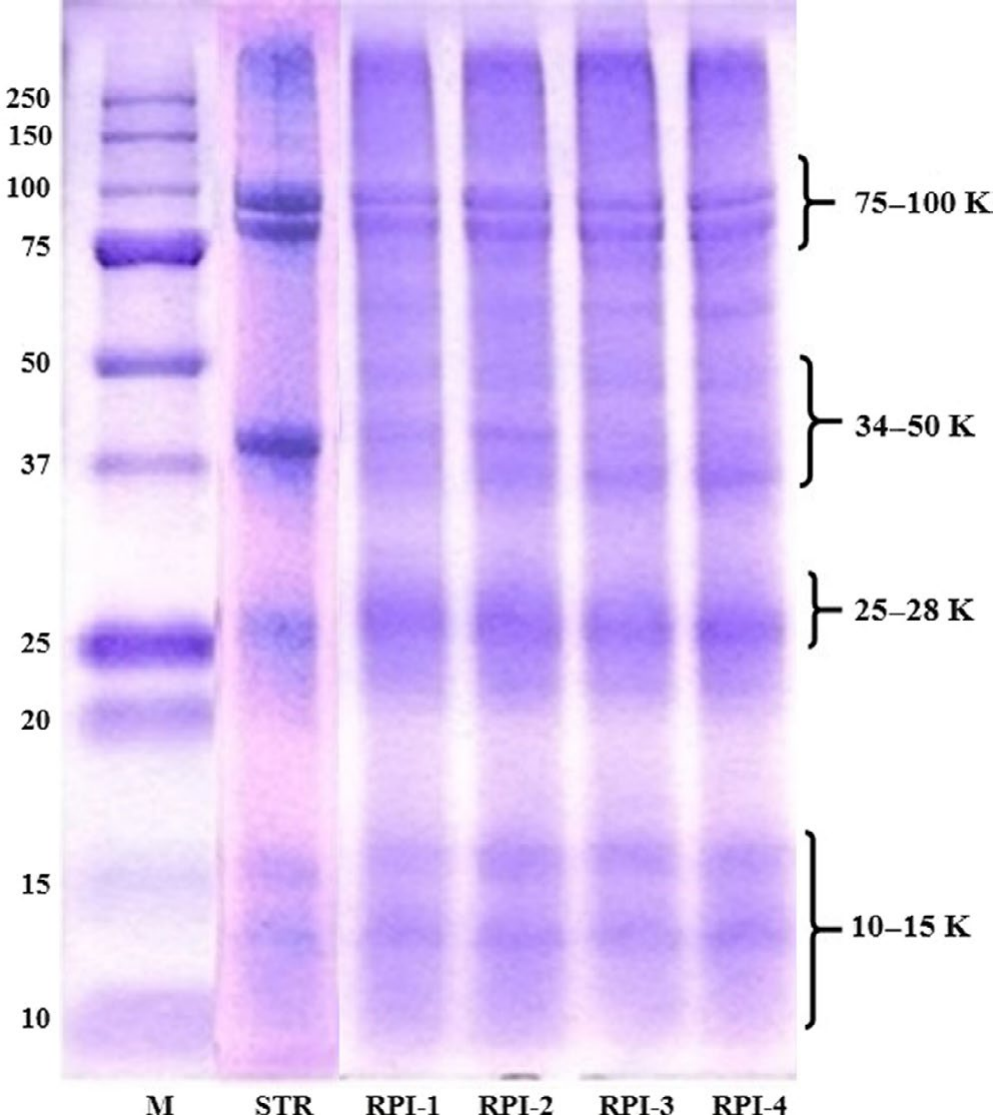
SDS‐PAGE patterns of protein isolates isolated from skipjack tuna roe (RPIs) using isoelectric solubilization and precipitation. M, protein maker; STR, skipjack tuna roe; RPI‐1, RPI‐2, RPI‐3, and RPI‐4, roe protein isolates adjusted to pH 4.5 and 5.5 after alkaline solubilization at pH 11 and 12

The SDS‐PAGE profiles of the STR and RPIs are shown in Figure 1. The major protein fractions of STR were observed at 100–75, 50–37, 25, 15, and 15–10 kDa. A dense protein band at 103 kDa was detected in the soluble and insoluble fractions of Alaska walleye pollock roe (Bechtel, Chantarachoti, Oliveira, & Sathivel, 2007), and four dense protein bands of 40–100‐kDa were observed in spray‐dried catfish protein powder (Sathivel et al., 2009). In the RPI protein bands, the 42‐kDa protein band of STR was absent and the 75–100‐kDa bands were weaker than those of STR. The disappearance of the 42‐kDa protein band indicates that this protein was not readily soluble during alkaline solubilization. The protein bands of acid‐precipitated RPIs after alkaline solubilization were 75–100, 25, and 10–15 kDa.

Al‐Holy and Rasco (2006) predicted that three protein bands in salmon caviar, with molecular weights of 96, 20, and 10 kDa, were vitellin‐like protein, lysozyme, and phosvitin, respectively. Additionally, a protein with a molecular weight of 27 kDa in Sturgeon's caviar may be an ovomucoid, which is generally a glycoprotein with a MW of 27–29 kDa (Al‐Holy & Rasco, 2006). Intarasirisawat et al. (2011) reported that protein bands of 32.5, 29, and 32.5 kDa were detected in skipjack, tongol, and bonito roes, respectively, which were ovomucoid or phosvitin.

### 3.3 Food functionalities

#### 3.3.1 Buffer capacity

**Figure 2:**
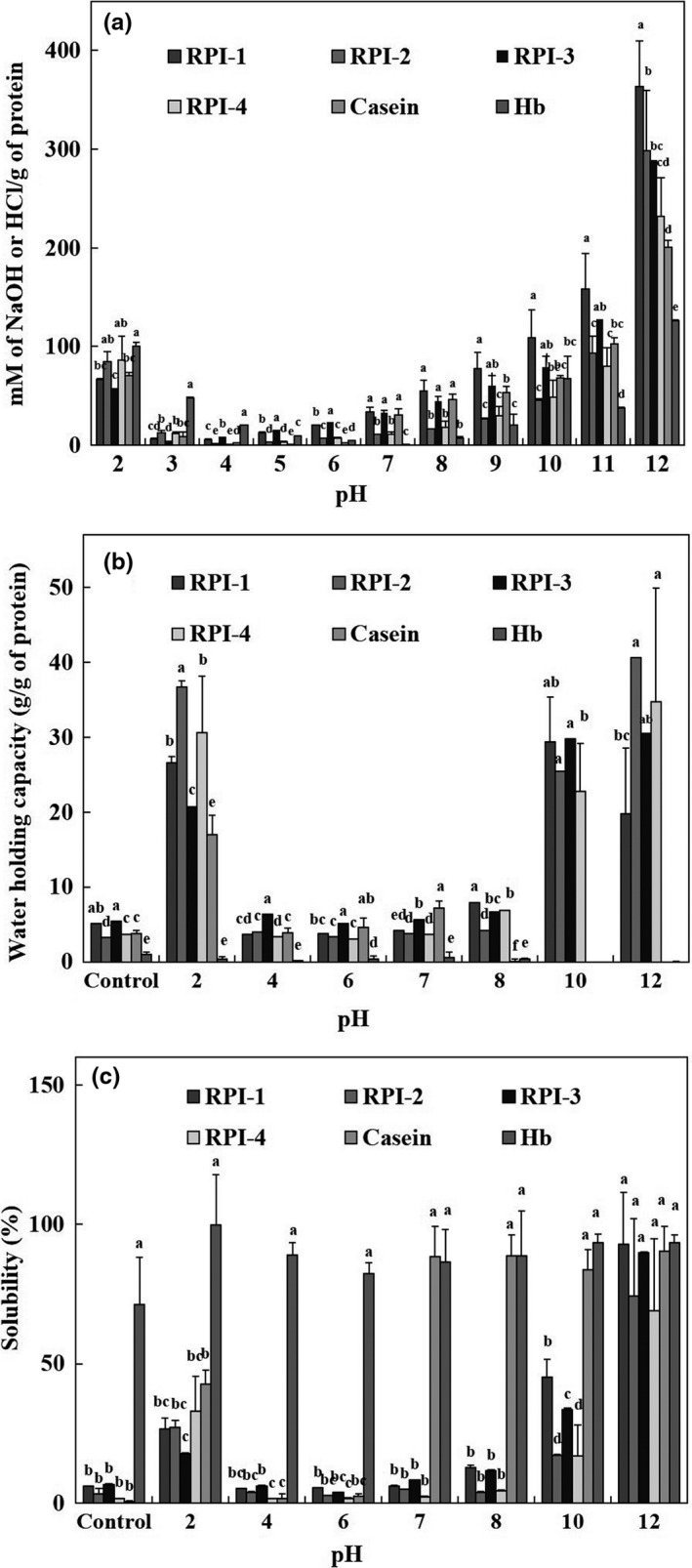
Buffer capacity (a), water‐holding capacity (b), and solubility (c) of protein isolates recovered from skipjack tuna roe (RPIs) using isoelectric solubilization and precipitation without (control) and with pH‐shift treatment. Values are expressed as the mean ± *SD* of triplicate determinations. Means with different letters within the sample and pH are significantly different at *p* < .05 by Duncan's multiple range test. Hb, hemoglobin

Buffer capacity is defined as the volume (ml) or amount (mmol) of HCl or NaOH required to induce a unit change in pH. The buffer capacities of the RPIs and positive controls are shown in Figure 2a. Over a pH range of 2–6, RPI‐4 required 23.2 mM HCl (average) to cause unit pH change, which is higher than that required by the other RPIs (19.9–22.8 mM HCl). In contrast, in the pH range of 6–12, RPI‐1 required a higher alkali concentration (63.9 mM NaOH) to cause a unit pH change compared with the other RPIs (39.9–51.8 mM NaOH). In comparison, casein and hemoglobin required 18.1 and 26.3 mM HCl (pH range 2–6) and 33.8 and 21.8 mM NaOH (pH range 6–12), respectively, to cause a unit pH change. These values were significantly lower than those required for the RPIs (*p* < .05). Therefore, the buffer capacities of the RPIs and casein were higher under alkaline conditions than under acidic conditions, showing a similar trend as roe concentrates of yellowfin tuna (Park et al., 2016). Chalamaiah, Balaswamy, Narsing Rao, Prabhakara Rao, and Jyothirmayi (2013) reported that dehydrated egg protein concentrate required an average of 0.65 mmol HCl and 1.22 mmol NaOH/g to induce a unit pH change under both acidic and alkaline conditions, which was higher than that for defatted egg protein concentrate. The lower buffer capacity of the dehydrated egg protein concentrate may be related to the presence of fatty components, which require more acid or alkali to induce a unit pH change.

Thus, RPIs recovered from STR were superior to those from other species and may resist changes in external pH. These RPIs with excellent buffering capacity can be used to develop protein‐fortified food components under different processing environments.

#### 3.3.2 WHC

Water‐holding capacity depends on protein–water interactions that affect protein function. Mohamed, Xia, Issoufou, and Qixing (2012) reported that interactions between proteins and water or oil are important in food systems, as they affect the flavor and texture of food. WHCs (g per g protein) with or without pH‐shift treatment (pH 2.0–12.0) of the RPIs and positive controls are shown in Figure 2b. In the control samples, the WHCs of RPI‐1 and RPI‐3 (5.1 and 5.4 g/g protein, respectively) were higher than those of RPI‐2, RPI‐4, and casein (3.3–3.8 g/g protein). After alkaline solubilization, the WHCs of RPI‐1 and RPI‐3 (recovered at pH 4.5) were significantly higher than those of RPI‐2 and RPI‐4 (recovered at pH 5.5). This may be because of the fractionation effect between the recovered RPIs during ISP. The WHC of solubilized hemoglobin (0.9 g/g protein) after pH‐shift treatment was significantly lower than those of the RPIs and casein (*p* < .05).

The WHCs of the RPIs were 20.7 ± 0.0–36.7 ± 0.9 g/g protein at pH 2 and 19.8 ± 8.8–40.6 ± 0.0 g/g protein at pH 10–12. Among the RPIs, RPI‐2 showed a relatively high WHC. In contrast, at pH 4–8, the WHC of the RPIs (3–6 g/g protein) was similar to that of the control. Thus, pH‐shift treatment significantly improved the WHC of RPIs at pH values other than pH 4–8, at which WHC was minimized because of increased electrostatic repulsion. Mohamed et al. (2012) reported that WHCs of protein isolates from tilapia were 2.63–2.51 g/g protein, which are lower than those of the RPIs found in this study. Azadian, Nasab, and Abedi (2012) reported that the WHC of minced fish reached a minimum near the isoelectric point (pH 4–6). Chalamaiah et al. (2013) reported that the lack of polar amino groups on the surface of protein molecules reduced the WHC because these polar groups are responsible for protein–water interactions. The WHC of RPIs from STR was superior to that of yellowfin tuna roe (Park et al., 2016), *Labeo rohita* fish egg (Balaswamy, Jyothirmayi, & Rao, 2007), and mrigal egg protein concentrates (Chalamaiah et al., 2013). Particularly, our results showed that structural changes in proteins were actively induced at extreme pH (pH 2 and pH 10–12), which increased the WHC by more than 5–8‐fold compared with the controls. This is because acidic and alkaline pH shifts cause protein conformational changes in the RPIs, allowing hydrophilic amino acids to easily access the surrounding water and increase the WHC.

#### 3.3.3 Protein solubility

Solubility is an important functional property of proteins, as it affects rheological, hydrodynamic, and surface activity characteristics. Protein solubility is also important in many protein‐based formulations (Park et al., 2016). The solubilities (%) of the RPIs with and without pH‐shift treatment are shown in Figure 2c. The solubilities of the control RPIs (1.7 ± 0.0–6.7 ± 0.3%) were significantly lower (*p* < .05) than that of hemoglobin (71.3 ± 16.8%). However, the solubility of casein was the lowest (0.4%), and the protein was nearly insoluble in a 1% dispersion. The solubility of pH‐shifted RPIs increased significantly at pH 2 (17.8 ± 0.3–33.1 ± 12.4%) compared with the controls. At pH 12, their solubilities ranged from 69.1 ± 25.8 to 92.9 ± 18.7%, indicating a higher rate of solubility increase at alkaline pH. After alkaline solubilization, the solubilities of RPI‐1 and RPI‐3 after acid precipitation at pH 4.5 were significantly higher than those of RPI‐2 and RPI‐4 recovered at pH 5.5. However, the solubility of hemoglobin (82.3 ± 4.0–99.9 ± 17.9%) was not affected by pH‐shift treatment (pH 2–12). Casein at pH 2 (42.7 ± 4.9%) and pH 7–12 (83.8 ± 7.2–90.3 ± 9.0%) showed significantly higher solubility than the controls. The RPIs and casein showed the lowest solubilities near the isoelectric point of pH 4–6 because of acid‐ and alkali‐limited protein solubilization. These results indicate that extreme pH changes (such as pH 2 and 12) affected protein solubility by exposing more charged and polar groups to the surrounding water (Kristinsson & Rasco, 2000). The pH‐dependent solubility of proteins is important in food functional properties and food system applications, particularly when the pH is below 4 or over 7, and is affected by protein–protein and protein–solvent interactions and the surface hydrophilic–hydrophobic balance (Gbogouri, Linder, Fanni, & Parmentier, 2004). The high solubility of fish proteins is important in food applications because it affects other functional properties such as foam and emulsification characteristics (Kristinsson & Rasco, 2000).

#### 3.3.4 FC and FS

Dispersions (1%, w/v) of the RPIs and positive controls were prepared in DDW to measure FC and FS. Before centrifugation, the FCs of 1%‐dispersed RPI‐1 and RPI‐3 (125.3 ± 2.2–128.6 ± 3.4%) were lower than that of hemoglobin (138.9 ± 18.3%), although the difference was not significant (*p* > .05), while RPI‐2, RPI‐4, and casein showed no FC. The FSs of RPI‐1, RPI‐3, and hemoglobin were relatively stable at 81.1 ± 8.0–88.0 ± 4.0±% for 60 min (Table 3). The FC and FS of the supernatant from dispersed samples after centrifugation with and without pH‐shift treatment are shown in Table 3.

**Table 3:** Foaming capacity (FC, %), foam stability (FS, %, 60 min), emulsifying activity index (EAI, m^2^/g of protein), and emulsion stability (ESI, min) with pH‐shift of protein isolates recovered from skipjack tuna roe (RPIs)

Sample		RPI‐1 (pH 11/4.5)	RPI‐2 (pH 11/5.5)	RPI‐3 (pH 12/4.5)	RPI‐4 (pH 12/5.5)	Casein	Hb
FC (%)	Control 1	128.6 ± 3.4^A^	100.0 ± 0.0^B^	125.3 ± 2.2^A^	100.0 ± 0.0^B^	100.0 ± 0.0^B^	138.9 ± 18.3^A^
	Control 2	147.0 ± 4.6^Ac^	100.0 ± 0.0^Cd^	135.8 ± 4.8^Ac^	100.0 ± 0.0^Cd^	109.4 ± 11.1^BCb^	120.9 ± 11.9^Ba^
	pH 2	191.3 ± 5.8^Ab^	185.1 ± 10.8^ABa^	159.5 ± 6.3^CDb^	168.7 ± 4.2^BCa^	140.3 ± 23.6^Dab^	115.1 ± 8.8^Ea^
	pH 4	148.0 ± 9.9^Ac^	100.0 ± 0.0^Dd^	137.8 ± 3.8^ABc^	100.0 ± 0.0^Dd^	109.1 ± 10.8^CDb^	125.2 ± 18.7^BCa^
	pH 6	144.0 ± 6.8^Ac^	100.0 ± 0.0^Cd^	139.8 ± 2.9^Ac^	100.0 ± 0.0^Cd^	107.0 ± 8.4^BCb^	126.1 ± 26.5^ABa^
	pH 7	128.8 ± 5.6^ABd^	118.6 ± 0.4^Bc^	145.8 ± 8.4^ABc^	114.2 ± 5.6^Bcd^	158.6 ± 37.0^Aab^	132.0 ± 17.1^ABa^
	pH 8	154.0 ± 2.8^ABc^	131.8 ± 0.6^ABbc^	145.3 ± 3.9^ABc^	120.5 ± 5.8^Bb^	184.8 ± 65.0^Aa^	139.8 ± 21.7^ABa^
	pH 10	197.6 ± 9.5^Aab^	128.2 ± 0.8^Bbc^	139.9 ± 0.9^Bc^	125.4 ± 2.2^Bb^	135.6 ± 12.8^Bab^	140.0 ± 22.5^Ba^
	pH 12	208.7 ± 7.6^Aa^	186.7 ± 13.0^Aa^	200.3 ± 14.8^Aa^	183.4 ± 25.0^Aa^	130.5 ± 9.7^Bab^	129.7 ± 7.8^Ba^
FS (%, 60 min)	Control 1	88.0 ± 4.0^A^	‐	86.1 ± 7.3^A^	‐	‐	81.1 ± 8.0^A^
	Control 2	‐	‐	‐	‐	‐	81.4 ± 3.4^a^
	pH 2	‐	28.4 ± 1.8^Bc^	‐	56.4 ± 4.9^Ab^	41.3 ± 9.8^ABb^	52.4 ± 12.2^Ac^
	pH 4	‐	‐	‐	‐	73.6 ± 3.1^Ba^	52.9 ± 7.5^Ac^
	pH 6	43.3 ± 6.8^Bb^	‐	‐	‐	76.9 ± 0.7^Aa^	76.0 ± 11.5^Aab^
	pH 7	68.9 ± 8.0^Aa^	‐	71.8 ± 3.0^Aa^	‐	74.4 ± 4.5^Aa^	74.5 ± 6.6^Aabc^
	pH 8	77.2 ± 8.0^Aa^	‐	73.7 ± 0.2^Aa^	65.4 ± 7.3^Aab^	43.1 ± 18.9^Bb^	63.0 ± 5.9^Aabc^
	pH 10	73.6 ± 6.3^ABa^	78.4 ± 6.2^Aa^	75.0 ± 2.6^Aa^	70.4 ± 4.5^ABa^	62.4 ± 1.7^ABa^	56.1 ± 20.7^Bbc^
	pH 12	69.2 ± 2.3^Aa^	59.1 ± 4.3^Ab^	60.5 ± 7.9^Ab^	61.7 ± 3.5^Aab^	69.1 ± 9.1^Aa^	57.1 ± 16.6^Abc^
EAI (m^2^/g protein)	Control 1	3.1 ± 0.1^BC^	1.8 ± 0.1^CD^	3.9 ± 0.5^B^	3.4 ± 0.2^BC^	0.4 ± 0.3^D^	18.4 ± 2.1^A^
	Control 2	14.7 ± 1.1^Be^	3.9 ± 0.9^De^	15.9 ± 2.1^Be^	2.3 ± 0.5^Dd^	7.0 ± 0.9^Ce^	19.1 ± 1.9^Aab^
	pH 2	15.5 ± 1.2^Be^	20.3 ± 1.4^Ac^	14.2 ± 0.9^Be^	19.2 ± 3.9^Ac^	11.9 ± 1.1^Bcd^	20.1 ± 1 2.3^Aab^
	pH 4	14.8 ± 0.9^ABe^	5.5 ± 0.1^Ce^	15.6 ± 2.9^Ae^	5.6 ± 0.3^Cd^	2.3 ± 0.5^Df^	12.9 ± 1.7^Bc^
	pH 6	13.3 ± 0.8^Ce^	9.8 ± 2.5^Dd^	9.2 ± 0.4^Df^	23.8 ± 1.2^Ac^	9.1 ± 1.7^Dde^	18.1 ± 1.3^Bab^
	pH 7	21.0 ± 0.2^ABd^	18.8 ± 1.8^Bc^	20.5 ± 0.4^Bd^	23.7 ± 1.7^Ac^	13.8 ± 2.6^Cc^	18.4 ± 1.8^Bab^
	pH 8	27.3 ± 0.9^Ac^	22.2 ± 1.0^Bc^	26.5 ± 1.7^Ac^	26.2 ± 1.3^Ac^	12.8 ± 1.3^Dc^	18.3 ± 0.5^Cab^
	pH 10	33.6 ± 2.4^Ab^	36.5 ± 0.7^Ab^	33.4 ± 2.7^Ab^	34.2 ± 5.4^Ab^	23.7 ± 2.7^Bb^	16.1 ± 4.4^Cbc^
	pH 12	54.2 ± 4.8^Aa^	54.0 ± 4.1^Aa^	55.0 ± 0.6^Aa^	53.8 ± 9.2^Aa^	36.8 ± 2.2^Ba^	20.8 ± 1.5^Ca^
ESI (min)	Control 1	‐	‐	‐	‐		20.0 ± 6.4^C^
	Control 2	30.7 ± 7.0^Abc^	‐	34.5 ± 4.6^Ab^	‐	31.8 ± 3.6^Ab^	20.1 ± 3.7^Bab^
	pH 2	15.3 ± 1.5^ABc^	16.9 ± 1.7^ABb^	17.0 ± 1.1^ABc^	20.2 ± 7.9^ABc^	13.2 ± 2.3^Bc^	21.2 ± 2.0^Aa^
	pH 4	16.7 ± 1.7^Cc^	56.2 ± 15.1^Ab^	17.2 ± 1.7^Cc^	29.1 ± 4.0B^Cc^	37.6 ± 13.9^Bb^	18.5 ± 2.1^Cab^
	pH 6	31.2 ± 3.7^Bbc^	61.0 ± 18.1^Ab^	55.3 ± 0.0^Ab^	53.1 ± 17.4^Ab^	23.5 ± 4.3^Bbc^	18.6 ± 1.2^Bab^
	pH 7	56.2 ± 4.5^Bab^	58.2 ± 8.1^ABb^	37.3 ± 4.3^Cab^	67.6 ± 8.8^Aab^	14.8 ± 1.7^Dc^	18.4 ± 2.7^Dab^
	pH 8	25.2 ± 2.9^Bbc^	70.5 ± 11.9^Ab^	23.6 ± 1.8^Bab^	63.4 ± 1.3^Aab^	16.0 ± 2.3^Bc^	18.3 ± 1.8^Bab^
	pH 10	27.5 ± 6.3^CDbc^	38.2 ± 5.5^Bb^	29.8 ± 3.3^BCb^	53.5 ± 7.3^Ab^	26.1 ± 6.9^CDbc^	18.0 ± 1.2^Dab^
	pH 12	83.4 ± 49.2^ABa^	127.2 ± 74.8^Aa^	114.3 ± 48.3^Aa^	83.8 ± 26.4^ABa^	62.7 ± 13.9^ABa^	16.6 ± 1.8^Bb^

Note

Controls 1 and 2 refer to the samples before and after centrifugation of the 1% dispersions, respectively. Values represent the mean ± *SD* of *n* = 3. Means with different capital letters within same row and small letters within the same column are significantly different at *p* < .05 by Duncan's multiple range test.

‐, not detected

The FCs of RPI‐1, RPI‐3, and casein increased by 147.0 ± 4.6%, 135.8 ± 4.8%, and 109.4 ± 11.1%, respectively, compared with the dispersed samples; however, RPI‐2 and RPI‐4 showed no foaming. The FC of hemoglobin decreased (138.9 ± 18.3% to 120.9 ± 11.9%) after centrifugation. During pH‐shift treatment (pH 2–12), the FCs of RPI‐1 and RPI‐3 was significantly increased at pH 2 (191.3 ± 5.8% and 159.3 ± 6.3%, respectively) and pH 10–12 (139.9 ± 0.9–208.7 ± 7.6%) compared with the controls (147.0 ± 4.6% and 135.8 ± 4.8%, respectively) (*p* < .05); however, FCs at pH 4–8 (128.8 ± 5.6–154.0 ± 2.8%) showed no significant difference (*p* > .05). The FCs of RPI‐2 and RPI‐4 were significantly increased at pH 2 (185.1 ± 10.8% and 168.7 ± 4.2%, respectively) and pH 7–12 (118.6 ± 0.4–186.7 ± 13.0%) compared with the controls, whereas no FC (100.0%) was observed at pH 4–6. The maximum FC of the RPIs (183.4 ± 25.0–208.7 ± 7.6%) and casein (184.8 ± 65.0%) were observed at pH 12 and 8, respectively. At pH 2–12, hemoglobin showed 115.1 ± 8.8–140.0 ± 22.5% FC; however, pH‐shift treatment did not induce a significant difference (*p* > .05). Regardless of pH‐shift treatment, the FCs of RPI‐1 and RPI‐3 were superior to those of RPI‐2, RPI‐4, casein, and hemoglobin. Proteins must rapidly migrate to the air–water interface, unfold, and rearrange at the interface to show good foam performance. Mutilangi, Panyam, and Kilara (1996) suggested that the foaming ability of proteins could be improved by increasing flexibility, which exposes a larger number of hydrophobic residues and reduces surface tension. The FS of hemoglobin (81.4 ± 3.4%) was maintained for up to 60 min in control 2 (without pH‐shift), but the RPIs and casein showed no FS (Table 3). The FSs of RPIs (59.1 ± 4.3–77.2 ± 8.0%) were more stable at pH 7–12 than at pH 2–6 until 60 min after whipping. Additionally, RPI‐1 and RPI‐3 were more stable than RPI‐2 and RPI‐4, and the foam characteristics of RPI‐1 were superior to those of RPI‐3. All samples showed a low foaming capacity at pH 4–6 because of their low WHC and solubility (Figure 2) near the isoelectric point, and FS depends on the degree of protein–water and protein–protein interactions within the matrix (Mutilangi et al., 1996).

#### 3.3.5 EAI and ESI

The oil‐in‐water EAI and ESI were determined to evaluate the ability of the samples to emulsify in foods such as soups, sauces, confectionery breads, and dairy products. The EAI (m^2^/g protein) and ESI (min) of the RPIs and positive controls are shown in Table 3. The EAI of the dispersed RPIs (control 1) was 1.8 ± 0.1–3.9 ± 0.5 m^2^/g protein, and RPI‐3 showed the highest EAI (3.9 ± 0.5 m^2^/g protein). However, the EAI of the RPIs was significantly lower than that of hemoglobin (18.4 ± 2.1 m^2^/g protein) (*p* < .05). After centrifugation (control 2), the EAI of the supernatant of the dispersed samples was generally increased, except for that of RPI‐4 (2.3 ± 0.5 m^2^/g protein). The EAIs of RPI‐1 (14.7 ± 1.1), RPI‐3 (15.9 ± 2.1), and casein (7.0 ± 0.9) were notably higher than those of the dispersions before centrifugation (control 1).

The ESI of hemoglobin (control 1) with an EAI of 18.4 ± 2.1 m^2^/g protein was 20.0 ± 6.4 min, while those of the RPIs and casein were undetectable. After centrifugation (control 2), there was no significant difference in the ESIs (30.7 ± 7.0–34.5 ± 4.6 min) of RPI‐1, RPI‐3, and casein (*p* > .05), although their ESIs were significantly higher than that of hemoglobin (20.1 ± 3.7 min) (*p* < .05). The increase in EAI and ESI after centrifugation may be related to the presence of insoluble particles in the dispersion that interfere with emulsion layer formation. EAI assessment of RPIs and positive controls after pH‐shift (pH 2–12) treatment showed that RPI‐2, RPI‐4, casein, and hemoglobin had the lowest EAIs (2.3 ± 0.5–12.9 ± 1.7 m^2^/g protein) at pH 4 and RPI‐1 and RPI‐3 had the lowest EAIs (13.3 ± 0.8 and 9.2 ± 0.4 m^2^/g protein, respectively) at pH 6 (Table 3). These results suggest that the FC and emulsifying activity are closely related to protein solubility in food, which was minimal in the RPIs and positive controls at pH 4–6. At pH 2 and 7–12, the EAIs of all samples, except for hemoglobin, were higher than that of control 2. At all pH ranges except for pH 2, the EAIs of RPI‐1 and RPI‐3 were higher than those of RPI‐2 and RPI‐4. The EAI of the RPIs showed no significant differences (*p* > .05) at pH 2 and 7–12; however, the emulsifying activity significantly increased with pH (*p* < .05). The emulsion stability did not increase in proportion to the increase in emulsifying activity with pH. The large deviation in emulsion stability after pH‐shift treatment may have occurred because of the nonuniformity of emulsified particles. Mutilangi et al. (1996) reported that a higher content of high‐MW peptides or hydrophobic peptides contributes to the stability of emulsions. Additionally, low‐MW peptides exhibit good emulsification properties, although they are not amphipathic. The emulsifying properties of proteins are largely affected by solubility, molecular size, surface hydrophobicity, net charge, steric hindrance, and molecular flexibility (Gbogouri et al., 2004).

### 3.4 Antioxidative and antihypertensive activity

Table 4 shows the ABTS^+^ radical scavenging activity (IC_50_, μg/ml), tyrosinase inhibitory activity (%), and ACE inhibitory activity (%) of RPI‐1. The ABTS^+^ radical scavenging assay can be performed on both lipophilic and hydrophilic compounds and has been widely used as an antioxidant activity assay. The ABTS^+^ radical scavenging activity (IC_50_) of RPI‐1 (1.5 ± 0.1 mg protein/ml) was 102.7 ± 1.0 μg/ml, which was better than those of alcalase and Protamex hydrolysates from shrimp processing byproducts (160 and 170 µg/ml, respectively) (Kim et al., 2016). UV irradiation can generate reactive oxygen species that influence skin pigmentation. Tyrosinase inhibitors have recently gained attention in the medical and cosmetic industries because of their association with hyper‐pigmentation (Choi, Kim, & Lee, 2011). The tyrosinase inhibitory activity of RPI‐1 was 13.5 ± 1.7%, and some whitening effects were predicted. Choi et al. (2011) reported a tyrosinase inhibitory activity of 31% for tuna cooking drip, but this value increased with the dose of gamma irradiation. Choi et al. (2017) reported that anchovy muscles not subjected to subcritical water hydrolysis inhibited tyrosinase activity by 14.65%. In these experimental results and reports, tyrosinase inhibitory activity was also observed in proteinous materials containing proteins or amino acids, but its inhibitory activity was low. The inhibition of ACE, a key enzyme that regulates blood pressure, has been recognized as an effective therapy for treating hypertension. The ACE inhibitory activity of RPI‐1 was 44.0 ± 4.9%. The current search for natural ACE‐inhibiting peptides has extended into seafood protein sources, particularly seafood byproducts. The hydrolysates of the byproducts of skin (Ngo, Ryu, & Kim, 2014) and yellow sole frame (Jung et al., 2006) showed 35%–86% ACE inhibitory activities, which are consistent with our results. These results suggest that RPIs from STR possess antioxidative and antihypertensive activities, which can be improved by enzymatic hydrolysis.

**Table 4:** ABTS^+^ radical scavenging activity, tyrosinase inhibitory activity, and angiotensin‐converting enzyme (ACE) inhibitory activity of roe protein isolate‐1 (RPI‐1)

Sample	Protein† (mg/ml)	ABTS^+^, IC_50_ (μg/ml)	Tyrosinase inhibitory activity (%)	ACE inhibitory activity (%)
RPI−1‡	1.5 ± 0.1	102.7 ± 1.0	13.5 ± 1.7	44.0 ± 4.9

Note

Values represent the mean ± *SD* of *n* = 3.

Abbreviations: IC_50_, the half maximal inhibitory concentration.

^†^ Based on the Lowry's (1951) methods.

^‡^ Supernatant of 1% dispersion after centrifugation.

## 4 CONCLUSION

The aims of this study were to investigate the physicochemical and functional properties of protein isolates recovered from skipjack tuna roe (STR) by ISP. The roe protein isolates (RPIs) were similar or superior to the positive controls (casein and hemoglobin) and other fish protein isolates in terms of buffering capacity, foaming ability, and emulsifying ability. The RPIs were rich in proteins containing essential amino acids and exhibited suitable functional characteristics for supplementing surimi‐based products and traditional foods. They also showed in vitro antioxidant and antihypertensive activities. This study suggests that high value‐added products can be developed from fish roes, which are currently underutilized in the seafood processing industry.

## CONFLICT OF INTEREST

The authors claim no conflicts of interest.

## ETHICAL APPROVAL

This study did not involve human or animal testing.
